# Editorial: Update on the diagnosis, treatment, and research of cerebral cavernous malformations

**DOI:** 10.3389/fneur.2026.1795369

**Published:** 2026-02-17

**Authors:** Yang Yang, Sheng Wang, Ruiqing Ni

**Affiliations:** 1 Department of Neurosurgery, Shanghai Sixth People's Hospital, Shanghai Jiao Tong University, Shanghai, China; 2 Department of Neurosurgery, Tongji Hospital, Wuhan, China; 3 Department of Nuclear Medicine, Inselspital, University of Bern, Bern, Switzerland; 4 Institute for Biomedical Engineering, ETH Zurich, Zurich, Switzerland

**Keywords:** biomarker, cerebral cavernous malformations, molecular mechanisms, neuroimaging, surgical management

Cerebral cavernous malformations (CCMs) represent a distinct and heterogeneous spectrum of cerebrovascular lesions composed of clusters of dilated, thin-walled capillaries devoid of intervening brain parenchyma. Clinically, CCMs may remain asymptomatic or manifest with debilitating complications such as intracranial hemorrhage, seizures, or progressive focal neurological deficits. The unpredictable natural history of CCMs, together with marked interindividual variability in lesion behavior, continues to pose major challenges for clinical management. Advances in molecular biology, neuroimaging, and therapeutic strategies have begun to reshape current paradigms in recent years (1). The articles featured in this Research Topic highlighted novel translational research in CCMs. These findings demonstrated critical roles of disease temporality, individualized risk stratification, and personalized treatment planning in shaping the future of cerebrovascular care.

The CCM_Italia cohort study protocol presented by Lanfranconi et al. presents a well-designed prospective protocol aimed at systematically characterizing the natural history of familial CCMs. Familial forms, driven by pathogenic variants in CCM-related genes, are often multifocal and associated with higher cumulative risks over a patient's lifetime. This multicenter registry integrates standardized clinical assessments, high-resolution MRI, and longitudinal blood sampling in both adult and pediatric patients, regardless of symptom status. By focusing on clinically meaningful endpoints, such as new symptomatic intracerebral hemorrhage or focal neurological deficits. The study is designed to identify clinical, radiological, and circulating biomarkers predictive of disease activity. Crucially, this protocol establishes a foundational framework for future risk-stratification models and offers a structured platform for translational research, addressing a long-standing unmet need in CCM investigation.

In parallel, Shi et al. explore treatment optimization for patients with surgically high-risk lesions using gamma knife radiosurgery. Their study demonstrates a significant reduction in post-treatment hemorrhage rates and identifies the biologically effective dose (BED) as an independent predictor of hemorrhage control, lesion regression, and favorable clinical outcomes. This study moves beyond traditional dose-based metrics by integrating radiobiological principles into radio-surgical planning. This offers a more sophisticated framework for optimizing the balance between therapeutic efficacy and safety. These insights are especially relevant for deep-seated or eloquent-area CCMs, where microsurgical resection involves significant risk and radiosurgery is an important therapeutic option.

Additionally, Wildi et al. address the knowledge gap and concerns regarding the safety of female hormone therapy in patients with CCMs. Hormonal therapies, in theory, may associate with pro-thrombotic risk. Micro-thrombosis has been shown to play a role in CCM symptomatology. The author found no significant increase in the risk of intracranial hemorrhage or focal neurological deficits in female hormone therapy users compared to non-users. These findings suggest a more individualized, evidence-based approach to hormone therapy for women with CCMs. As the currently study has a limited cohort size, future multicenter studies with detailed hormonal dosing data and biomarker monitoring are warranted.

The systematic review and meta-analysis article by Liu et al. focuses on the role of oxidative stress-related insulin-like growth factor-1 (IGF-1) in ischemic stroke. Elevated IGF-1 was found to be associated with poorer functional outcomes 1 year post-stroke (Liu et al.). Although circulating IGF-1 levels are not linked to the acute risk of ischemic stroke or short-term functional outcomes, IGF-1 is found to be more related to long-term neural remodeling, inflammation, and vascular recovery than to acute injury. These findings suggest that IGF-1 has the potential as indicator of extended recovery trajectories post-stroke. Further study is needed to elucidate whether IGF-1 can inform long-term rehabilitation or be a therapeutic target for chronic stroke.

In summary, translating research into precise, patient-centered care will be critical for the CCM management. Combining long-term registry data, with clinical, and biomarker insights, will improve our understanding of dynamic risk profiles. This will help to improve prognostic accuracy, predict individual disease trajectories, and enable precision medicine strategies for each patients.
